# Editorial: Lung Imaging in Respiratory Failure

**DOI:** 10.3389/fphys.2022.862647

**Published:** 2022-03-03

**Authors:** Lorenzo Ball, Patricia R. M. Rocco, Paolo Pelosi

**Affiliations:** ^1^Department of Surgical Sciences and Integrated Diagnostics (DISC), University of Genoa, Genoa, Italy; ^2^Anesthesia and Intensive Care, Ospedale Policlinico San Martino, IRCCS per l'Oncologia e le Neuroscienze, Genoa, Italy; ^3^Laboratory of Pulmonary Investigation, Carlos Chagas Filho Institute of Biophysics, Federal University of Rio de Janeiro, Rio de Janeiro, Brazil

**Keywords:** computed tomography, ARDS, lung imaging, electrical impedance tomography (EIT), lung ultrasonography (LUS), respiratory failure, intensive care

The last two decades have seen increasing interest toward delivering personalized treatments to patients admitted to the intensive care unit (ICU) with acute respiratory failure (ARF), in particular acute respiratory distress syndrome (ARDS) (Pelosi et al., 2021). However, identifying simple clinical characteristics allowing targeted respiratory support and other treatments is challenging. Lung imaging techniques are capable of describing different phenotypes of lung injury as well as the effects of ventilatory support on the respiratory system (Ball et al., 2017b), and have shown promising results as tools to guide mechanical ventilation strategies in ARDS (Constantin et al., 2019). Moreover, research in the field of imaging applied to respiratory failure has been boosted by the ongoing COVID-19 pandemics, providing valuable clinical information that helped clinicians in the timely development of appropriate strategies in such a rapidly evolving scenario (Grasselli et al., 2020; Ball et al., 2021a). This editorial summarizes the articles enclosed in this Frontiers Research Topic “Lung Imaging in Respiratory Failure.”

An introductory review highlights how different imaging techniques depict specific pathophysiological aspects of the lungs and how their quantitative analysis can provide functional information on the respiratory system (Musch). Despite being widely used, each imaging technique has specific methodological pitfalls that require elucidation in the future. A systematic review focusing on lung ultrasound (LUS) explored its diagnostic accuracy in discriminating different patterns of lung injury (Yuan et al.). Several lung imaging techniques were often used to measure or estimate lung recruitment, namely the amount of non-aerated lung that can be aerated following changes in ventilator settings or recruitment maneuvers. A systematic review covering studies using LUS, computed tomography (CT), and electrical impedance tomography (EIT) concluded that the estimation of lung recruitment based on lung imaging techniques is poorly standardized and that the ability of imaging techniques to predict lung recruitment in ARDS remains uncertain (Pierrakos et al.). On the other hand, a simple scoring system of chest X-ray, the Radiographic Assessment of Lung Edema, showed good diagnostic accuracy for identifying patients with ARDS according to the Berlin definition (Zimatore et al.).

Several papers in this Research Topic focused on bedside imaging techniques such as EIT and LUS. In an interesting experimental study, authors propose a sophisticated analysis of EIT-derived parameters, combined with airway pressure data, to derive information concerning transpulmonary pressure, exploiting the relationships between respiratory mechanics partitioning and regional heterogeneity of ventilation in ARDS (Scaramuzzo et al.). Such estimate might provide important information to improve the understanding of respiratory support in ARDS, in a context where the esophageal pressure monitoring is still underused in the clinical practice (Akoumianaki et al., 2014). In addition to its role in adult chest imaging, LUS has an established role in the pediatric setting, also for the particularly favorable acoustic window that characterizes these patients. In a review covering 10 years of research in this field, benefits, limitations, and possible future challenges of pediatric LUS are discussed (Musolino et al.). In another research article, the efficiency of deep learning and artificial intelligence to classify lung ultrasound images in the pediatric setting is explored with good results (Magrelli et al.).

Another application of artificial intelligence proposed in this article collection is semi-automated segmentation of lung images obtained with CT. This high-resolution technique is a recognized standard for the assessment of lung aeration (Pesenti et al., 2016) as well as lung recruitment in ARDS (Gattinoni et al., 2006) and COVID-19 (Ball et al., 2021b), but manual delimitation of lung is time consuming (Reske et al., 2010; Ball et al., 2017a). Computer-based approaches using neural networks performed acceptably in two original research papers when applied to both human and experimental animal CT scans with different lung findings, including in repeated scans aimed at measuring lung recruitability (Herrmann P., et al.; Maiello et al.).

In other research papers included in this Research Topic, lung imaging techniques and their derived parameters were used as endpoints to assess the effects of specific changes in respiratory support strategies. In an experimental study in pigs receiving one-lung ventilation and thoracic surgery, lateral compared to supine positioning was associated with higher relative perfusion, regardless of the presence of intravascular hypovolemia (Wittenstein et al.). Another research paper investigated the dependency on positive end-expiratory pressure (PEEP) of patients receiving non-invasive respiratory support for COVID-19-related ARF; the authors observed, using EIT, that lung de-recruitment during a PEEP-decrease trial was associated with failure of non-invasive respiratory support (Rauseo et al.). These findings might improve the understanding of the role of non-invasive respiratory support in COVID-19, while avoidance of intubation is often feasible but in certain patients associated with a high risk of developing self-inflicted lung injury (Battaglini et al., 2021). In another study, the effects of PEEP were studied in invasively ventilated brain injured critically ill patients using quantitative CT and assessing the effect on intracranial pressure (Robba et al.). In an experimental study using dynamic four-dimensional computed tomography, the authors explored the role of inhomogeneity in determining the expiratory kinetics of gases in different lung regions, highlighting how poorly aerated regions might be particularly susceptible to ventilator-induced lung injury (Herrmann J., et al.).

Finally, two papers used imaging techniques addressed specific controversies regarding the concept of mechanical power. This parameter was proposed as a parameter to guide mechanical ventilation parameters in patients with ARDS (Gattinoni et al., 2016; Silva et al., 2019). However, several aspects concerning its calculation remain controversial. In a model of ARDS, a correlation between mechanical power and neutrophilic inflammation was confirmed using positron emission tomography (Scharffenberg et al.). However, in another experimental study using CT and EIT, mechanical power was reduced with the decrease in the respiratory rate alone, while maintaining constant CO_2_ levels through the use of extracorporeal membrane oxygenation at increasing gas flows. The reduction in respiratory rate worsened lung atelectasis despite reducing mechanical power (Spinelli et al.).

We are grateful to the authors and reviewers that contributed to this Research Topic, covering a wide range of interesting and challenging aspects of innovative applications of lung imaging techniques of respiratory failure from research to clinical practice.

## Author Contributions

LB, PR, and PP drafted and revised this editorial. All authors contributed to the article and approved the submitted version.

## Conflict of Interest

The authors declare that the research was conducted in the absence of any commercial or financial relationships that could be construed as a potential conflict of interest.

## Publisher's Note

All claims expressed in this article are solely those of the authors and do not necessarily represent those of their affiliated organizations, or those of the publisher, the editors and the reviewers. Any product that may be evaluated in this article, or claim that may be made by its manufacturer, is not guaranteed or endorsed by the publisher.
